# HEALTH CARE USE AND OUT-OF-POCKET COSTS FOR RURAL FAMILY CAREGIVERS AND CARE RECIPIENTS IN TRANSITIONAL PALLIATIVE CARE

**DOI:** 10.1093/geroni/igae098.2113

**Published:** 2024-12-31

**Authors:** Brystana Kaufman, Ro Huang, Diane Holland, Catherine Vanderboom, Jay Mandrekar, Allison Gustavson, Joan Griffin

**Affiliations:** Duke University School of Medicine, Durham, North Carolina, United States; Duke University, Durham, North Carolina, United States; Mayo Clinic, Rochester, Minnesota, United States; Mayo Clinic, Rochester, Minnesota, United States; Mayo Clinic, Rochester, Minnesota, United States; Minneapolis Veterans Affairs Health Care System, Minneapolis, Minnesota, United States; Mayo Clinic, Rochester, Minnesota, United States

## Abstract

Few studies have reliable estimates on costs of informal care following hospitalization, including health care use and out-of-pocket costs for family caregivers (FCGs) and their care recipients (CR). This presentation discusses results from a Transitional Palliative Care (TPC) intervention on healthcare use and out-of-pocket spending for both FCGs and CRs. The study used data from the Technology-Enhanced TPC study, a trial comparing the TPC intervention to support FCGs of seriously ill CRs to an attention control condition. TPC FCGs received teaching, guidance, and counseling via video calls for 8 weeks following CR hospital discharge. After discharge, a research assistant called FCGs once a month for up to 6 months or CR death to collect healthcare utilization (e.g. outpatient, emergency department, and hospital), out-of-pocket healthcare spending (e.g. deductibles and coinsurance), and health-related travel costs (e.g., transportation, lodging, food) for FCGs and CRs. Incidence rate ratios (IRR) were estimated using negative binomial regressions. The study included 282 FCG-CR dyads across 3 US states. TPC reduced nights in the hospital for CR (IRR = 0.75; 95% CI = 0.56 – 0. 99). Total out-of-pocket spending was not significantly different for TPC versus control; however, TPC dyads reported lower lodging costs (IRR = 0.71; 95% CI = 0.56-0.89). Across groups, mean out-of-pocket spending for dyads was $1401.85, with healthcare payments contributing $1048.58 and transportation expenses contributing $136.79. This study contributes to evidence that palliative care interventions reduce unnecessary hospitalization for seriously ill patients, yet the financial costs to caregivers remain high.

